# Successful treatment of desmoid tumor of the chest wall with tranilast: a case report

**DOI:** 10.1186/1752-1947-4-384

**Published:** 2010-11-29

**Authors:** Takahiro Goto, Tetsuo Nemoto, Koichi Ogura, Takahiro Hozumi, Nobuaki Funata

**Affiliations:** 1 Department of Orthopaedic Surgery and Musculoskeletal Oncology, Tokyo Metropolitan Cancer and Infectious Diseases Center Komagome Hospital, 3-18-22 Hon-Komagome, Bunkyo-ku, Tokyo 113-8677, Japan; 2 Department of Pathology, Tokyo Metropolitan Cancer and Infectious Diseases Center Komagome Hospital, 3-18-22 Hon-Komagome, Bunkyo-ku, Tokyo 113-8677, Japan

## Abstract

**Introduction:**

Desmoid tumor is characterized by infiltrative growth and local recurrence often occurs after surgery. To reduce the local recurrence rate, adjuvant therapy, such as radiotherapy and pharmacotherapy with cytotoxic agents, anti-estrogen agents and non-steroidal anti-inflammatory drugs, is often applied. In addition, these non-surgical treatments are also performed in patients with unresectable desmoid tumors. We successfully treated a patient with a desmoid tumor with tranilast; an anti-allergic agent.

**Case presentation:**

A 48-year-old Japanese man with a slow-growing desmoid tumor on his chest wall was treated with an oral administration of tranilast (300 mg per day, three times a day). Two years and two months after the commencement of his therapy, the tumor became impalpable. At this time, the oral administration of tranilast was discontinued. Two years after discontinuation of the treatment, a physical examination showed no recurrence of the tumor and he continued in a state of remission. We were successfully able to reduce the size of the tumor and thereafter maintain the reduced size.

**Conclusion:**

Tranilast was clinically effective in our case, and is probably comparable to cytotoxic agents or anti-estrogen agents. Because tranilast has substantially fewer adverse effects than cytotoxic agents, it could be a very useful therapeutic agent for desmoid tumor.

## Introduction

Desmoid tumor, defined as clonal fibroblastic proliferation, arises in the deep soft tissue and is characterized by infiltrative growth and a tendency towards local recurrence but without the ability to metastasize. The standard and definitive treatment for desmoid tumor is wide excision, and adjuvant radiotherapy is sometimes applied. Although neither distant metastasis nor malignant transformation occurs, the reported local recurrence rate after excision is high: the local recurrence rate today is approximately 50 percent [1]. Pharmacotherapy, with cytotoxic agents, anti-estrogen agents or non-steroidal anti-inflammatory drugs (NSAIDs), is performed in addition to radiotherapy for both recurrent tumors and unresectable tumors. The efficacy of the pharmacotherapy is variable but its adverse effects are significant. Therefore, a possible new kind of pharmacotherapy with minimal adverse effects is desirable.

Desmoid tumors and keloids have common histological features where fibroblasts proliferate abnormally and abundant collagen fibers deposit locally. We hypothesized that tranilast, a therapeutic agent for keloid and hypertrophic scars, might be effective for desmoid tumors. We report the case of a patient with desmoid tumor on his chest wall whom we treated with tranilast. We were successfully able to reduce the size of the tumor and thereafter maintain the reduced size. To the best of our knowledge, there has been no reported case of desmoid tumor treated with tranilast.

## Case presentation

**Figure 1:**
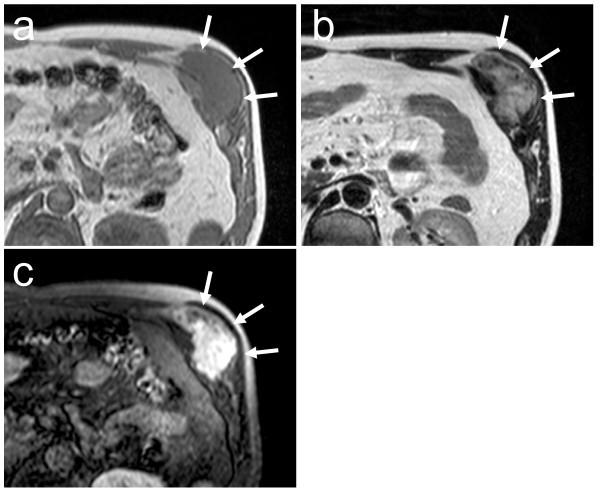
**Axial MRI of the chest wall at his first visit.** T1-weighted image (a) shows isosignal intensity area, whereas T2-weighted image (b) shows high intensity area. The tumor is strongly enhanced on gadolinium-enhanced T1-weighted image (c). Arrows indicate the tumor.

**Figure 2:**
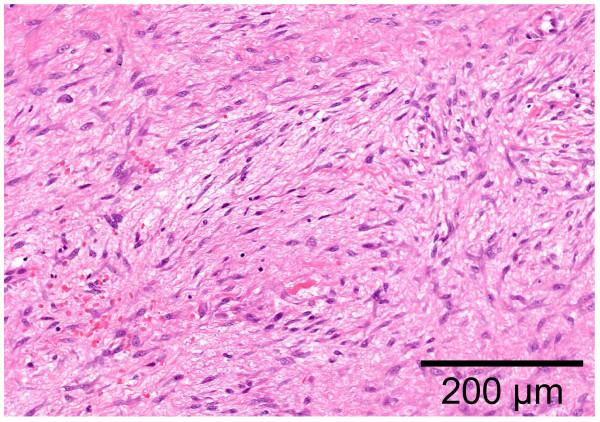
**Photomicrograph of the biopsy specimen: spindle or stellate-shaped cells are uniformly proliferating in a collagenous stroma with no nuclear atypia (hematoxylin and eosin stain)**. Scale bar indicates 200 μm.

**Figure 3:**
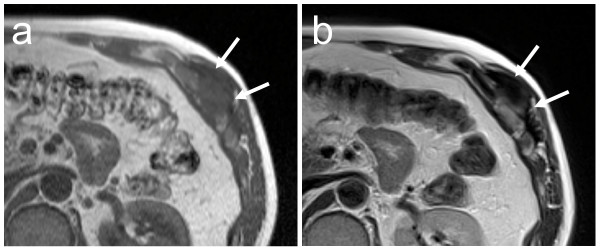
**Axial MRI of the chest wall after treatment with tranilast.** The tumor on T1-weighted image (a) is smaller than that seen on his first visit (shown in Figure 1(a)). The proportion of low-signal intensity area on T2-weighted image (b) is larger than that seen on his first visit (shown in Figure 1b). Arrows indicate the tumor.

A 48-year-old Japanese man noticed a slightly tender soft tissue mass on the anterolateral aspect of the lower part of his left chest wall, six months prior to his first visit to our hospital. When the tumor became slightly enlarged, he visited his previous orthopedist. A soft tissue tumor was suspected and an incisional biopsy was performed. However, a definite pathological diagnosis could not be made due to the small amount of biopsy specimen. He was referred to our hospital for further examination and treatment. On inspection, the anterolateral surface of the lower part of his left chest wall was swollen. On palpation, a fibrously hard tumor, measuring 5 × 6 cm, was palpable. It adhered firmly to the chest wall. He was experiencing mild pain. There was no localized heat or redness. Chest radiographs showed no abnormal findings. A magnetic resonance imaging (MRI) scan showed that the soft tissue tumor (measuring 6 × 5.5 × 5 cm) was located beneath his external oblique muscle and permeated into his intercostal muscles, causing the peritoneum to protrude towards his abdominal space (Figure 1a, b). The signal intensity of the lesion was isointense to skeletal muscle on T1-weighted images (Figure 1a) and heterogeneously high on T2-weighted images with some areas of low intensity (Figure 1b). The lesion was strongly enhanced by the administration of a contrast medium (Figure 1c). As a result of these physical and MRI findings, we suspected a soft tissue tumor rich in collagen fibers and blood vessels, such as desmoid tumor, fibrosarcoma or malignant fibrous histiocytoma. He was unwilling to take a second biopsy and a natural course was observed. The tumor gradually enlarged, becoming 8 × 6 cm on palpation ten months after his first visit. At this time, a second incisional biopsy was performed. Macroscopically, the tumor was whitish and fibrous. Histologically, the tumor was poorly circumscribed and infiltrated into his surrounding soft tissue. Spindle or stellate-shaped cells of uniform appearance proliferated in a collagenous stroma (Figure 2). The nuclei were slightly irregular but lacked hyperchromasia or atypia and had small nucleoli. The mitotic rate was less than 1 per 10 high power field (HPF). From these histological findings, we made the diagnosis of desmoid tumor. A wide excision of the tumor, combined with his ribs and peritoneum, was planned. However, he refused to undergo surgery. In addition, he also refused to undergo radiotherapy and chemotherapy with cytotoxic agents. As an alternative, we commenced pharmacotherapy with tranilast (Rizaben^®^, Kissei Pharmaceutical Co., Matsumoto City, Japan). Tranilast was administered orally every day (300 mg per day, three times a day). Immediately after commencement of the therapy, the size of the tumor did not change and the tumor seemed stabilized. Six months after initiation of the therapy, the tumor size began to decrease. Two years and two months after commencement of the therapy, the tumor became impalpable. At this time, the oral administration of tranilast was discontinued. Two years after the discontinuation of treatment, a physical examination showed no recurrence of the tumor and he continued in a state of remission. An MRI revealed that the tumor still existed, but it was shrunken in size and the protrusion of the peritoneum towards his abdominal space seen on his first visit was no longer evident (Figure 3a, b). T2-weighted images (Figure 3b) showed that the proportion of low-signal intensity area was much larger than that seen on his first visit, suggesting a decrease in the tumor's cellular component.

## Discussion

Desmoid tumor is a slow-growing, permeative tumor, uniformly composed of fibroblasts and abundant collagen. Desmoid tumor of the abdominal wall mostly occurs in women during pregnancy or soon after delivery, whereas extra-abdominal desmoid tumor is more frequent from puberty to 50 years of age, with both men and women equally affected. The principal site of extra-abdominal desmoid tumor is the musculature of the shoulder, followed by the chest wall and back, the thigh, and then the head and neck [2].

The conventional treatment for extra-abdominal desmoid tumor is surgery. If practical, surgical excision with a wide margin is the definitive treatment for desmoid tumors. The high recurrence rate after excision is partly due to an inadequate surgical margin for tumors of such an infiltrative nature [1]. When excision with an adequate surgical margin is impractical, adjuvant radiotherapy is considered [3]. When the tumor is unresectable because of its location or size, radiotherapy may be performed. Because radiotherapy can induce secondary sarcoma, and because desmoid tumor occurs mainly in patients under 40 years of age, it should be avoided if possible. Like radiotherapy, chemotherapy may be performed after excision as an adjuvant therapy or as a main therapy for unresectable tumors. Treatment with chemotherapy has been reported in combination with various cytotoxic agents; low-dose methotrexate and vinblastine [4], cyclophosphamide and doxorubicin [5], mitomycin, doxorubicin and cisplatin [5] and ifosfamide and etoposide [5]. NSAIDs, such as indomethacin and sulindac, are also effective. Although there are a high number of case reports where NSAIDs were effective in the treatment of desmoid tumor, there have been only a few reports where their efficacy was discussed in relation to a significant number of patients [6]. Estrogen receptor has been found to exist in desmoid tumors, and anti-estrogen therapy with tamoxifen has been reported to be effective in some patients [7]. It has been reported that combined pharmacotherapy with sulindac and tamoxifen is effective in 70 percent of patients with desmoid tumor [8]. However, cytotoxic agents have significant adverse effects and the selection of patients for treatment is important. Likewise, anti-estrogen agent is potentially hazardous to endocrine systems, especially in premenopausal patients. If NSAIDs are administered for a long period, they often cause peptic ulcers and renal disturbances. Therefore, a new modality of pharmacotherapy with significantly fewer adverse effects is a desirable option.

In our case report, tranilast was clinically very effective and probably comparable to cytotoxic agents or to anti-estrogen agents. Not only was the tumor size reduced, but the proportion of high-signal intensity area on a T2-weighted MRI also became much smaller after the treatment with tranilast. Presumably, a second-look biopsy would reveal a reduction in the tumor's cellular component. Tranilast was originally developed as a therapeutic agent for bronchial asthma. In addition, it is effective for allergic rhinitis, atopic dermatitis and allergic conjunctivitis. It acts in these allergic diseases by inhibiting the release of chemical inflammatory mediators, such as prostaglandin E2 (PGE2) and histamine, from mast cells. Besides these allergic diseases, it is also effective for keloid and hypertrophic scars. It has been demonstrated that fibroblasts in keloid and hypertrophic scars produce much more transforming growth factor-β1 (TGF-β1) than normal fibroblasts [9]. TGF-β1 is known to stimulate collagen synthesis [10]. Tranilast suppresses collagen synthesis in keloid or hypertrophic scars, which is mediated by inhibiting the release of TGF-β1 from fibroblasts [10]. On the other hand, PGE2 and histamine released from mast cells stimulate the proliferation of fibroblasts [11,12]. Tranilast inhibits their release from mast cells [13,14], which in turn inhibits the proliferation of fibroblasts. Thus, the inhibitory mechanism of keloid or hypertrophic scar formation by tranilast is achieved by suppressing the proliferation of fibroblasts and by inhibiting collagen synthesis. Likewise, fibroblasts in desmoid tumor have been proven to produce much more TGF-β1 than normal fibroblasts [15]. By comparison with keloid and hypertrophic scars, it is natural to presume that tranilast should inhibit collagen synthesis and the proliferation of fibroblasts in desmoid tumor, leading to shrinkage of the tumor. Since tranilast has fewer adverse effects, it is superior to the other reported pharmacotherapies. In addition, tranilast is administered orally. In the case of chemotherapy with cytotoxic agents, weekly, bi-weekly or monthly hospital visits or prolonged hospitalization is necessary for the treatment. By contrast, a course of treatment with tranilast requires less frequent hospital visits with no need for prolonged hospitalization.

Although we cannot determine the efficacy of tranilast solely from our case report, tranilast seems to be a potentially desirable therapeutic agent for desmoid tumor. Further study with a higher number of patients with desmoid tumor is needed. If the efficacy of this drug is demonstrated in the future, we will not only use it as a first-line adjuvant therapy for surgery with inadequate surgical margins, but also as a first-line therapy for unresectable tumors.

## Conclusion

Pharmacotherapy with tranilast was effective in a patient with desmoid tumor on his chest wall. Because tranilast has substantially fewer adverse effects than cytotoxic agents, anti-estrogen agents and NSAIDs, it could be a useful therapeutic agent for desmoid tumor.

## Abbreviations

HPF: high power field; NSAIDs: Non-steroidal anti-inflammatory drugs; PGE2: prostaglandin E2; TGF-β1: transforming growth factor-β1.

## Consent

Written informed consent was obtained from the patient for publication of this case report and any accompanying images. A copy of the written consent is available for review by the Editor-in-Chief of this journal.

## Competing interests

The authors declare that they have no competing interests.

## Authors' contributions

TG, KO and TH were the orthopedic surgeons who performed the biopsy and close follow-up of the patient. TN and NF were the pathologists who performed the histological examination and made the histological diagnosis of the tumor. TG and TN drafted the manuscript. All authors read and approved the final manuscript.

## References

[B1] Ballo MT Zagars GK Pollack A Pisters PW Pollock RA Desmoid tumor: prognostic factors and outcome after surgery, radiation therapy, or combined surgery and radiation therapy J Clin Oncol 1999 17 158 167 10458229 10.1200/JCO.1999.17.1.158

[B2] Weiss SW Goldblum JR Weiss SW, Goldblum JR Benign fibroblastic/myofibroblastic proliferations Enzinger and Weiss's Soft Tissue Tumors 2008 5 St. Louis: Mosby 175 225

[B3] Jelinek JA Stelzer KJ Conrad E Bruckner J Kliot M Koh W Laramore GE The efficacy of radiotherapy as postoperative treatment for desmoid tumors Int J Radiat Oncol Biol Phys 2001 50 121 125 10.1016/S0360-3016(00)01570-4 11316554

[B4] Azzarelli A Gronchi A Bertulli R Tesoro JD Baratti D Pennacchioli E Dileo P Rasponi A Ferrari A Pilotti S Casali PG Low-dose chemotherapy with methotrexate and vinblastine for patients with advanced aggressive fibromatosis Cancer 2001 92 1259 1264 10.1002/1097-0142(20010901)92:5<1259::AID-CNCR1446>3.0.CO;2-Y 11571741

[B5] Okuno SH Edmonson JH Combination chemotherapy for desmoid tumors Cancer 2003 97 1134 1135 10.1002/cncr.11189 12569616

[B6] Klein WA Miller HH Anderson M DeCosse JJ Use of indomethacin, sulindac, and tamoxifen for the treatment of desmoid tumors associated with familial polyposis Cancer 1987 60 2863 2868 10.1002/1097-0142(19871215)60:12<2863::AID-CNCR2820601202>3.0.CO;2-I 2824015

[B7] Kinzbrunner B Ritter S Domingo J Rosenthal CJ Remission of rapidly growing desmoid tumors after tamoxifen therapy Cancer 1983 52 2201 2204 10.1002/1097-0142(19831215)52:12<2201::AID-CNCR2820521204>3.0.CO;2-# 6640490

[B8] Hansmann A Adolph C Vogel T Unger A Moeslein G High-dose tamoxifen and sulindac as first-line treatment for desmoid tumors Cancer 2004 100 612 620 10.1002/cncr.11937 14745880

[B9] Younai S Nichter LS Wellisz T Reinisch J Nimni ME Tuan TL Modulation of collagen synthesis by transforming growth factor-beta in keloid and hypertrophic scar fibroblasts Ann Plast Surg 1994 33 148 151 10.1097/00000637-199408000-00005 7979045

[B10] Suzawa H Kikuchi S Arai N Koda A The mechanism involved in the inhibitory action of tranilast on collagen biosynthesis of keloid fibroblasts Jpn J Pharmacol 1992 60 91 96 10.1254/jjp.60.91 1282576

[B11] Topol BM Lewis VL Jr Benveniste K The use of antihistamine to retard the growth of fibroblasts derived from human skin, scar, and keloid Plast Reconstr Surg 1981 68 227 232 10.1097/00006534-198108000-00018 6114504

[B12] Durant S Duval D Homo-Delarche F Effect of exogenous prostaglandins and nonsteroidal anti-inflammatory agents on prostaglandin secretion and proliferation of mouse embryo fibroblasts in culture Prostaglandins Leukot Essent Fatty Acids 1989 38 1 8 10.1016/0952-3278(89)90140-3 2608699

[B13] Koda A Nagai H Watanabe S Yanagihara Y Sakamoto K Inhibition of hypersensitivity reactions by a new drug, N(3',4'-dimethoxycinnamoyl) anthranilic acid (N-5') J Allergy Clin Immunol 1976 57 396 407 10.1016/0091-6749(76)90054-3 131140

[B14] Komatsu H Kojima M Tsutsumi N Hamano S Kusama H Ujiie A Ikeda S Nakazawa M Mechanism of inhibitory action of tranilast on the release of slow reacting substance of anaphylaxis (SRS-A) in vitro: effect of tranilast on the release of arachidonic acid and its metabolites Jpn J Pharmacol 1988 46 53 60 10.1254/jjp.46.53 2452913

[B15] Locci P Bellocchio S Lilli C Marinucci L Cagini L Baroni T Giustozzi G Balducci C Becchetti E Synthesis and secretion of transforming growth factor-beta1 by human desmoid fibroblast cell line and its modulation by toremifene J Interferon Cytokine Res 2001 21 961 970 10.1089/107999001753289578 11747628

